# Real-Time Vehicle Roll Angle Estimation Based on Neural Networks in IoT Low-Cost Devices

**DOI:** 10.3390/s18072188

**Published:** 2018-07-07

**Authors:** Javier García Guzmán, Lisardo Prieto González, Jonatan Pajares Redondo, Mat Max Montalvo Martínez, María Jesús L. Boada

**Affiliations:** 1 Computer Science and Engineering Department, Institute for Automotive Vehicle Safety (ISVA), Universidad Carlos III de Madrid, Avda. de la Universidad 30, 28911 Leganés, Madrid, Spain; lpgonzal@inf.uc3m.es (L.P.G.); mamontal@pa.uc3m.es (M.M.M.M.); 2 Mechanical Engineering Department, Institute for Automotive Vehicle Safety (ISVA), Universidad Carlos III de Madrid, Avda. de la Universidad 30, 28911 Leganés, Madrid, Spain; jopajare@ing.uc3m.es (J.P.R.); mjboada@ing.uc3m.es (M.J.L.B.)

**Keywords:** real-time estimation, IoT, artificial neural network, vehicle dynamics, roll angle, low cost devices, Raspberry Pi 3 Model B, Intel Edison, FANN

## Abstract

The high rate of vehicle-crash victims has a fatal economic and social impact in today’s societies. In particular, road crashes where heavy vehicles are involved cause more severe damage because they are prone to rollover. For this reason, many researches are focused on developing RSC Roll Stability Control (RSC) systems. Concerning the design of RSC systems with an adequate performance, it is mandatory to know the dynamics of the vehicle. The main problem arises from the lack of ability to directly capture several required dynamic vehicle variables, such as roll angle, from low-cost sensors. Previous studies demonstrate that low-cost sensors can provide data in real-time with the required precision and reliability. Even more, other research works indicate that neural networks are efficient mechanisms to estimate roll angle. Nevertheless, it is necessary to assess that the fusion of data coming from low-cost devices and estimations provided by neural networks can fulfill hard real-time processing constraints, achieving high level of accuracy during circulation of a vehicle in real situations. In order to address this issue, this study has two main goals: (1) Design and develop an IoT based architecture, integrating ANN in low cost kits with different hardware architectures in order to estimate under real-time constraints the vehicle roll angle. This architecture is able to work under high dynamic conditions, by following specific best practices and considerations during its design; (2) assess that the IoT architecture deployed in low-cost experimental kits achieve the hard real-time performance constraints estimating the roll angle with the required calculation accuracy. To fulfil these objectives, an experimental environment was set up, composed of a van with two set of low-cost kits, one including a Raspberry Pi 3 Model Band the other having an Intel Edison System on Chip linked to a SparkFun 9 Degrees of Freedom module. This experimental environment be tested in different maneuvers for comparison purposes. Neural networks embedded in low-cost sensor kits provide roll angle estimations highly approximated to real values. Even more, Intel Edison and Raspberry Pi 3 Model B have enough computing capabilities to successfully run roll angle estimation based on neural networks to determine rollover risk situations, fulfilling real-time operation restrictions stated for this problem.

## 1. Introduction

The high rate of vehicle-crash victims has a fatal economic and social impact in today’s societies. That is why current road vehicles incorporate safety systems in order to reduce accidents. In particular, road crashes where heavy vehicles are involved cause more severe damage because they are prone to rollover. For this reason, many researches are focused on developing Roll Stability Control (RSC) systems.

Concerning the design of RSC systems with an adequate performance, it is mandatory to know the dynamics of the vehicle. One of the most important parameters related to rollover dynamics is the roll angle. The problem is that this angle cannot be measured directly using low-cost sensors, so that it is necessary to estimate it through the integration and processing of data acquired from low-cost devices or from the sensors installed on current vehicles (sensor fusion) [1,2]. In previous works, roll angle is estimated using different sensor types: inertial angle sensor and a gyroscope [1], angular rate and accelerometer sensors [2], lateral accelerometers and gyroscope [3,4,5], lateral and longitudinal accelerometers and yaw rate and roll rate sensors [4,6,7,8], on-board vehicle sensors and low-cost GPS [9,10] and lateral tire force sensors [11]. These observers are based on Kalman filter [5,6,9,10,11], robust estimators [2,3,7,8] or artificial intelligence techniques [6,12].

The design of RSC systems is a complex task as they have to fulfill some requirements, mutual to other safety vehicle systems:

- To acquire information from sensors that have a high sampling frequency.
- To process sensor information in hard real time.
- To include actuators with fast-response time.
- To use low-cost systems in order to minimize the implementation cost in commercial vehicles.
- To develop an architecture that integrates all previous elements, guaranteeing high reliability and fault-tolerance.

The increase of computing power, the reduction of consumption and electric devices size, along with the high variety of communication technologies and networking protocols using the Internet have brought about development of Internet of Things (IoT), which is being applied nowadays not only in smart manufacturing, healthcare, and smart cities; but also in transportation and smart vehicles [13,14,15,16,17,18]. In addition, some research works have focused on hardware and software architectural problems related to this trend, and applied to the vehicular environment described before [19,20,21,22].

With the objective of design small and low-cost on-board systems for vehicle applications [19,20,21,22], it is necessary that they have enough accuracy and small processing time to increase vehicle safety by the inclusion of both estimators and controllers. These small computers should not only acquire data but also process it to estimate study variables. Raspberry Pi 3 Model B and Intel Edison are two popular small single-board computers, because they offer flexibility, low price and high support from the internet community. There are studies that use these systems like a processing device [23]; in [24], a fusion data for autonomous and transportation systems was performed through a Raspberry Pi. In [25], Raspberry Pi is used for detecting E. coli in real time. In [26], the dynamics of a human-powered vehicles was acquired through a Raspberry Pi. Finally, in [27], a study about inherent capabilities of the Raspberry Pi was carried out.

Like Raspberry Pi, there are many studies that use Intel Edison for the same purposes, although it is not a single-board computer. In [28], integration with biomedical devices are used to acquired real-time vital parameters on neonates. In [29], a prototype to analyze geospatial data was created with Intel Edison. In [30], a system for smart home based on Intel Edison is proposed.

Previous studies demonstrate that the previous low-cost devices can provide data in real-time with the required precision and reliability [23].

On the other hand, Artificial Neural Networks (ANN) have been used to estimate vehicular characteristics in previous studies; like [31], where an ANN is used to estimate truck static weights by fusing weight-in-motion data, [32] where an ANN is used to estimate friction coefficient of wheel and rail in trains, [33] where an ANN is used to predict intersection crashes, or [34] where an ANN is used to estimate the traffic density and vehicle classification.

With the increase of computational power in small and embedded devices, ANNs have become computationally feasible to be used in such systems. This enhances the capabilities that IoT devices can provide [35,36,37]. However, in most cases, it is necessary to assess that the fusion of data coming from low-cost devices and estimations provided by ANNs can fulfil the reliability and appropriateness requirements for using these technologies to improve overall safety in production vehicles.

There is a lack of pre-existing research work that provides data regarding several relevant questions related to embed ANN estimators in low-cost devices. These questions are:

- Do low-cost experimental kits using ANN to estimate the roll angle have enough performance to address the hard real-time processing constraints of at least 50 Hz?
- Considering that the kits satisfy the hard real-time constraints, are the estimations provided precise enough to identify rollover situations? Are these estimators sensitive to noise in real driving situations?
- What are the key lessons learnt to consider when implementing this kind of estimators in low-cost kits?

A relevant scientific contribution of this research work consists of providing experimental data to discuss these questions, addressing the lack of enough related research works. Additional contributions provided by this article are:

(a) Design and develop an IoT-based architecture, integrating ANN in low cost kits with different hardware architectures in order to estimate under real-time constraints the vehicle roll angle. This architecture is able to work under high dynamic conditions, by following specific best practices and considerations during its design.
  The IoT based architecture has been developed integrating low-cost Inertial Motion Unit (IMU) and small single-board computer that acquire data from the IMU sensor and estimate the roll angle using ANNs. The outcome to the estimations have been compared with the measurements acquired by a high-end professional device (VBOX from Racelogic), used as the ground truth. Two different low-cost systems have been considered on this research systems (Raspberry Pi 3 Model B with IMU BNO0055 and Intel Edison with IMU LSM9DSO). These devices are compared in terms of estimation accuracy, processing time and reliability.
(b) Assess that the IoT architecture deployed in low-cost experimental kits achieve the hard real-time performance constraints, estimating the roll angle with the required calculation accuracy. Even more, the noise influence in real driving situations is analyzed in order to evaluate the accuracy of the estimations provided.

This work is part of a research initiative that aims to design a full control system to improve lateral stability of commercial vehicles implementing an IoT architecture composed of pluggable interconnected low-cost intelligent sensors and actuators. Several results obtained in this research initiative are presented in [6,23,38]. Vargas-Meléndez et al. [6] propose the definition of a neural network to estimate roll angle using sensor fusion. The results obtained in that article were fully based on simulations created in CarSim and it was not experimented in real settings. In [23], an evaluation of precision and performance of low-cost kits to directly measure several variables (roll rate and lateral acceleration) that are essential to manage rollover risks situations is carried out. In [38], the implementation of a roll angle estimator based on Kalman filters is provided.

This article is structured as follows. In Section 2, the methodology is presented, including the experimental testbed design, experiments’ definitions, and the data gathering and analysis. The experimental results and the calculation of the RMS error and processing time are presented in Section 3. Finally, in Section 4, the discussion and conclusion of the results and the method are exposed.

## 2. Methodology

This section is organized in four subsections. First, it provides a description of the experimental testbed design designed; second, the objectives stated for this research work are enumerated and the experiments defined to achieve these goals are presented; third, the data gathering and analysis mechanisms are introduced; and finally, the threats to validity are briefly discussed.

### 2.1. Experimental Testbed Design

This research work experimental testbed design can be analyzed from two perspectives: hardware and software.

#### 2.1.1. Hardware Perspective

The experimental testbed is designed following the principles stated for Internet of Things (IoT) architectures. This testbed is packaged in a product that can be integrated in any vehicle, in this particular case, for testing purposes, a Mercedes-Benz van was used. The motivation to use this vehicle was to compare the results obtained during this research with those described in [6].

To properly perform the comparative analysis, three experimental kits were deployed:

- The reference, or ground truth kit, is composed of an Inertial Measurement Unit (IMU) from Racelogic connected to VBOX 3i GPS dual antenna data logger in a 90-degree angle with respect to the traveling direction [39]. These sensors are connected to a laptop embedded in the vehicle [6]. The installed sensors provide measurements for lateral acceleration, a_ym_, longitudinal acceleration, a_x_, yaw rate $\dot{\psi }$, roll rate $\dot{\varphi }$ and roll angle *φ*. Given the nature of Racelogic VBOX devices, they need to be physically connected by wire to the experiments manager and among themselves.
- The first low-cost experimental kit is based on a Raspberry Pi 3 Model B [40,41], including a low-cost Inertial Measurement Unit Shield [42].
- The second low-cost experimental kit consists of an Intel Edison System-on-Chip [43] linked to a SparkFun “9 Degrees of Freedom” module [44].

This network is provided by a MikroTIK Router Board, connected to the Internet. Moreover, the devices perform a time synchronization using the NTP protocol at the very beginning, to provide coherent timestamps in the sampled and processed results.

The technical specifications of hardware elements considered for ground truth, Raspberry and Intel Edison kits are detailed in Table 1.

**Figure 1:**
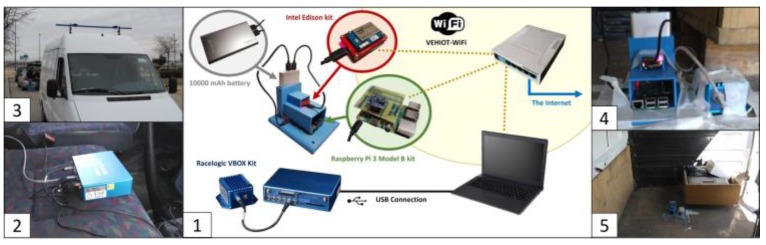
Hardware and connectivity (**1**); Racelogic VBOX data logger (**2**); van equipped with GPS dual-antenna (**3**); low-cost kits and Racelogic VBOX IMU (**4**,**5**).

The low-cost experimental kits and the IMU of the ground truth kit were located in the vehicle’s center of mass, as is depicted in Figure 1. All the kits were also interconnected using a WiFi router, which handles the communications among them, so the experiments can be synchronized, and the outcome of the tests can be gathered. According to [45], the accurate positioning of controller and IMU is essential for precision enhancement of low-cost kits.

#### 2.1.2. Software Architecture

The Internet of Things (IoT) refers to the interconnection of uniquely-identifiable embedded devices within the Internet infrastructure [45].

In the scope of this research work, according to the definition provided by [46], the IoT can be viewed as “a global infrastructure for the information society, enabling advanced services by interconnecting (physical and virtual) things based on existing and evolving interoperable information and communication technologies (ICT)”.

The main elements to consider in an IoT architecture are [22]:

(a) The perception layer that collects data using sensors. These elements constitute the most important ingredients of the IoT. In the scope of the experimental testbed designed, the “things” are the different low-cost experimental kits that can be installed in a vehicle to monitor risk rollover situations; they are able to communicate among themselves if complex vehicles are considered.
(b) The communications component is the next architectural element to be included in an IoT architecture. The most common communication technologies for vehicular communications are Bluetooth, Zigbee, and WiFi. In this research work, this component is considered through a WiFi connection through a wireless (802.11 g) access point.
(c) The last architectural component integrates two kinds of software elements: middleware and applications. These components are considered in the components enhancing the interoperability of smart things included in the testbed architecture.

**Figure 2:**
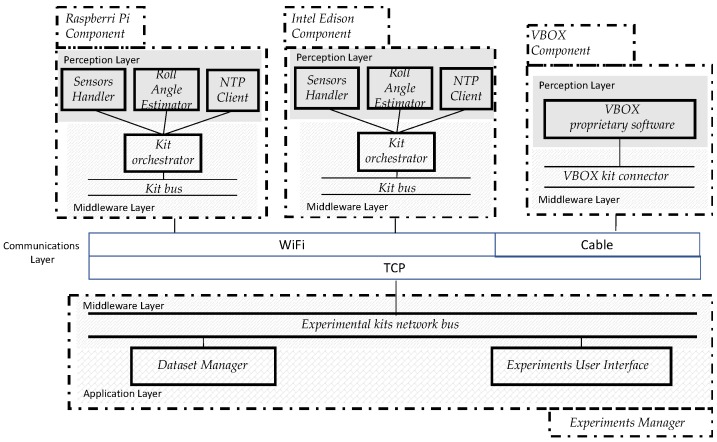
Testbed software design.

Figure 2 shows these key elements of an IoT architecture in the testbed designed.

A software architecture was designed to provide real-time roll angle values (ground truth kit) estimations (low-cost experimental kits) using yaw rate, roll rate, longitudinal and lateral acceleration values obtained from the sensors included in each experimental kit. This architecture provides in a synchronized way the data required to analyze the accuracy and performance to obtain roll-angle estimations. The main components of this architecture are shown in Figure 2.

The *Experiments Manager* is in charge of providing a user interface to let the researcher start and stop the experiments and register the information coming from the experimental kits. It is developed in C++. This component includes the following classes:

- The *experimental kits network bus* is in charge of subscribing and unsubscribing the different experimental kits. Even more, it provides the possibility to send requests to the experimental kits (0, shutdown experimental kit; 1, keep running the experiment; 2, start the experiment; and 3, end the experiment) and to receive the information items provided by VBOX, Raspberry Pi 3 Model B and Intel Edison kits.
- *Dataset Manager* is in charge of receiving the data coming from the kits and storing them in CSV files.
- *Experiments User Interface* that provide the functionality to start and finish the experiments. It connects to the *experimental kits network bus* to start and finish an experiment in a synchronized way for all the experimental kits connected.

The *VBOX Component* is in charge of managing the experiments execution using the Racelogic IMU sensor and GPS dual antenna data. It is developed in C#. The specific classes included in this component are:

- The *VBOX kit connector* oversees publishing of the experimental kit, receiving orders from the experimental kits orchestrator connected and sending to it information obtained during the experiment.
- The *VBOX proprietary software* is in charge of managing the information received during the experiment execution.

The software to manage the Intel Edison experimental kit is in charge of managing the information that the sensors included in its hardware architecture provide. This component is implemented in C++. The specific classes included in this component are:

- The *kit bus* that is in charge of publishing the experimental kits in the network, receiving the requests form *experimental kits network bus* and sending to the *kit orchestrator* the orders for starting and stopping the experiment.
- The *kit orchestrator* is in charge of integrating in a synchronized way the information provided by the sensors included in the kit. To achieve this aim, the orchestrator completes the following process: (1) When it receives the “start experiment” signal from the *kit bus*, it creates an empty data structure to store the results in RAM memory; (2) when the orchestrator receives the “end experiment” signal, it sends to the *Experiments Manager* the data structure, which included the data obtained during the experiment; then (3) *Experiments Manager* is in charge of storing this data. The information sent is routed through the *kit bus* and the *experimental kits network bus* to reach the *Dataset Manager* in the *Experiments Manager.*
- The *Sensors Handler* has the responsibility of registering data coming from sensors in a 50 Hz sampling rate.
- The *Roll Angle Estimator* is a software component that implements an ANN to estimate the roll angle corresponding to the lateral acceleration, a_ym_, the longitudinal acceleration, a_x_, the yaw rate $\dot{\psi }$, and the roll rate $\dot{\varphi }$ as input variables. A more detailed description of this estimator is provided in 2.1.3.
- The *NTP Client* is in charge of registering the actual date-time in the hardware controller of the experimental kit to ensure that all the kits in the testbed have the same date-time. This enables and eases comparison of results during the data analysis stage in this research work.

The Raspberry Pi 3 Model B and the Intel Edison have the same class structure. Sensor drivers were developed in C++ due to the recommendations provided in [23], and trying to keep the code as much similar as possible to maximize objectivity in the comparison of performance results against other devices with different hardware architectures (as the Intel Edison).

Intel Edison and Raspberry Pi 3 Model B kits include wireless communication interfaces (IEEE 802.11n) to facilitate connectivity with the Experiments Manager. The communication is managed and operated using TCP sockets. This configuration facilitates the installation of the low-cost experimental kits in any location inside the vehicle without communication wires. Even more, the sensors considered for low-cost experimental kits are straightforwardly attached to the development boards by using the GPIO ports.

#### 2.1.3. Vehicle Roll Angle Estimator Using Neural Networks

**Figure 3:**
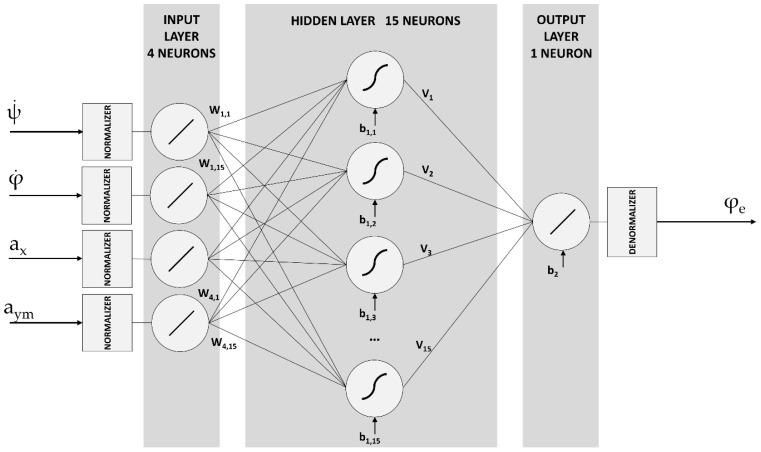
Artificial neural network architecture.

The vehicle Roll Angle Estimator component uses ANNs to estimate the vehicle roll angle. Most of the previous proposed estimators are based on model [1,2,3]. The main drawback of these algorithms is that they need detailed physical characteristics of the system in order to obtain an accurate model. To tackle this problem, Artificial Intelligence is now being used. The proposed ANN architecture used to estimate the vehicle roll angle was previously presented in [6]. The ANN consists of three layers as depicted in Figure 3: (1) an input layer; (2) a hidden layer and (3) an output layer. The number of neurons of the hidden layer is 15. The output of the ANN is the estimated vehicle roll angle, *φ_e_*, which is obtained as:

$$\varphi_{e}=g_{2}\left(\overset{15}{\underset{k=1}{\sum }}\left(v_{k}o_{k}\right)+b_{2}\right)$$

where $v_{k}$ are the weights between the hidden layer and output layer, $b_{2}$ is the bias in the output layer and $g_{2}$ is the linear activation function. $o_{k}$ is the output of the k-th neuron in the hidden layer, which is obtained as:

$$o_{k}=g_{1}\left(\overset{4}{\underset{l=1}{\sum }}\left(w_{lk}i_{l}\right)+b_{1l}\right)$$

where $w_{lk}$ are the weights between the input layer and the hidden layer, $b_{1l}$ are the biases in the hidden layer, $g_{1}$ is the tanh activation function and $i_{l}$ are the inputs of the ANN: (1) the lateral acceleration, a_ym_; (2) the longitudinal acceleration, a_x_; (3) the yaw rate, $\dot{\psi }$ and (4) the roll rate $\dot{\varphi }.$ These inputs are directly measured by the low-cost sensors. In [6], the Backpropagation algorithm was used for weights and biases adjustment. At each iteration step:

$$\theta \left(n+1\right)=\theta \left(n\right)-\eta \frac{\partial E}{\partial \theta }+\alpha \left[\theta \left(n\right)-\theta \left(n-1\right)\right]$$

where θ represents the weights and biases, n is the iteration number, η is the learning-rate, α is the momentum constant used for accelerating the learning process and E is the error error calculated as:

$$E={\left(\varphi_{d}-\varphi_{e}\right)}^{2}$$

where $\varphi_{d}$ is the desired response. The input-output patterns used to train the ANN during the learning phase were obtained from an experimentally-validated TruckSim vehicle model. Different maneuvers (double lane change, lane change and J-turn) under different speeds and road friction coefficients were simulated, so that the input-output patterns are representative and could characterize the non-linear vehicle behavior. The training data need to be normalized properly in order to achieve the best performance of the network.

In the proposed algorithm, the vehicle roll angle is estimated in each sample by using the information obtained directly from sensor signals, without integrating any signal and, for this reason, there is not accumulated error. The output of the ANN only depends on inputs and is not time-dependent.

In [6], ANN architecture was implemented in MATLAB code. The significant difference provided by this research work in comparison to [6] is the implementation of the ANN and its integration in the IoT based architecture, able to satisfy the real-time restrictions related to embed this estimator in a control unit installed in a real commercial vehicle. The ANN is implemented in C++ using the FANN framework [47] and cross-compiled for both low-cost kit architectures (ARM and x86).

Several experiments with different levels and neurons configurations were carried out in our previous research work [6], concluding that the proposed configuration is the most appropriate.

### 2.2. Experiments Definition

According to the goals stated for this research work, the hypotheses defined are:

**H1:**  The roll angle estimated (φ_e_) by the low-cost experimental kits is similar to the roll angle provided (φ_a_) by the ground truth kit (i.e., VBOX-based kit).

**H2:**  The low-cost experimental kits’ performance (i.e., Raspberry Pi 3 Model B and Edison Kits) estimating the roll angle achieves the levels required for real-time processing (50 Hz, forced by the sample rate of the low cost sensors [23]) embedded in operating vehicles.

To assess **H1**, a comparison among the roll angle estimated by Raspberry Pi 3 Model B kit, Intel Edison kit, a computer using the data from the Racelogic VBOX IMU as input, and the roll angle directly measured by Racelogic VBOX GPS dual antenna (ground truth) was carried out. The objective of using a computer to estimate the roll angle by means of the ANN estimator fed with the data captured from VBOX IMU is to determine whether the error comes from the ANN based estimator or from the low-cost devices.

To assess **H2**, the ANN estimation execution time per sample must be inferior to 20 ms (50 Hz) in both low-cost device kits.

Three controlled experiments were performed (see Table 2): a normal circulation driving (id 1), J-Turn (id 2) and lane change (id 3). To assess the validity of the results, every experiment was repeated three times, except for the case of the normal circulation driving test.

**Figure 4:**
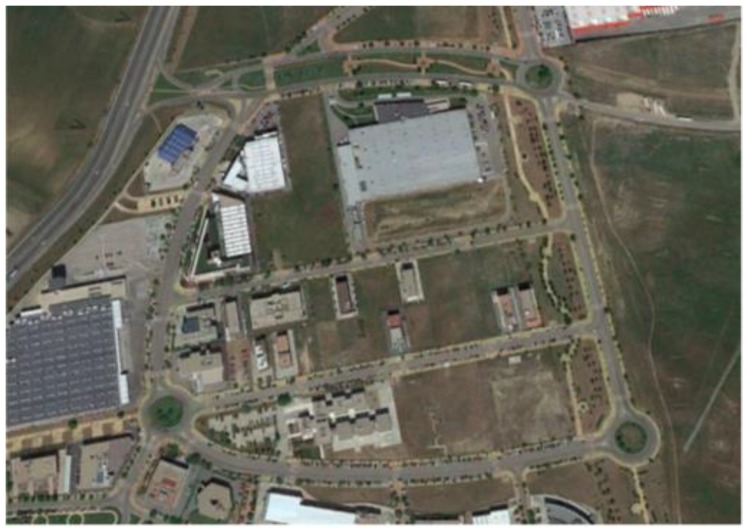
Experiments’ context (Map scale 1:7800 cm).

As shown in Figure 4, the considered tests were executed in Leganes (Madrid, Spain) using a Mercedes Sprinter van. The experiments were carried out when the road conditions were free from traffic restrictions preventing the correct execution of the considered maneuvers.

### 2.3. Data Gathering and Analysis

The data obtained for the previously-defined experiments (see Table 2) were stored by the controller of each experimental kit in a CSV formatted file, whose name included the type of experiment and its execution timestamp. The variables considered were lateral acceleration, a_ym_, longitudinal acceleration, a_x_, yaw rate, $\dot{\psi }$, roll rate, $\dot{\varphi }$, roll angle *φ*_a_ (only obtained in VBOX kit, that acts as the ground truth, together with the GPS coordinates) and the estimated roll angle, *φ_e_* (calculated by low-cost sensor kits and VBOX kit).

The accuracy of roll angle estimation using ANN and data collected by low-cost sensor kits was calculated comparing these data against the roll angle obtained from GPS-dual antenna by the Racelogic VBOX (Ground Truth). Section 3 presents the results obtained in the experiments defined for this research work.

### 2.4. Threats to Validity

Several threats were considered during the experiments’ definition to analyze the validity of the results obtained in this experimental work:

**(A) Internal validity**, which refers to the appropriate experiments definition preventing the introduction of systematic errors influencing results and conclusions. In this research, this kind of validity is related to the errors in sensors configuration, bugs in software components implemented to manage the experimental kits and the possible errors related to the maneuvers execution for each type of experiment. These threats were mitigated in the following way:

- The first threat was mitigated using two different units for each low-cost experimental kits (Intel Edison and Raspberry Pi 3 Model B) in order to prevent errors from hardware components working wrongly. Even more, all the units were verified in static conditions, implementing the corresponding calibrations, to ensure that experimental kits were providing appropriate values.
- Specific unit testing suites were defined and implemented to verify that the components included in the software architecture properly process the values obtained from sensors and synchronize correctly the information provided by each experimental kit.
- The ANN based estimators were properly trained and compared with information coming from experiments carried out in previous research works [6,23].
- To verify the validity of the results, each maneuver was repeated, at least, three times consecutively.

**(B) External validity**, which refers to the replication of the considered experiments and the generalization of the results obtained. In this research, this kind of validity is related to the type of sensors and controllers considered, the vehicle conditions, and the road conditions. These threats were mitigated in the following way:

- Regarding the type of sensors and controllers considered, the research team decided to use controllers and sensors having average features [48,49,50]. In this sense, due to the expected technologies improvement, the conclusions can be applied in forthcoming low-cost sensors.
- Regarding vehicle conditions, the experimental kits location is the most relevant threat to an appropriate generalization of the results obtained. To mitigate this threat, according to [23], the IMU and the low-cost experimental kits were located in the vehicle’s center of mass.
- Regarding experiments execution and road conditions, the road considered for the experiments exaction does not have a relevant slope. Even more, the experiments were executed with different types of directions, and constant and variable speed.

## 3. Results

As is indicated in Section 2, a Mercedes Sprinter van was used for this work (see Figure 1). Three different kind of experiments were carried out: two different maneuvers, J-Turn and lane change; and a normal circulation test. For J-Turn and lane change maneuvers, three similar tests were performed in order to assess the validity of the results.

### 3.1. Test 1. J-Turn

**Figure 5:**
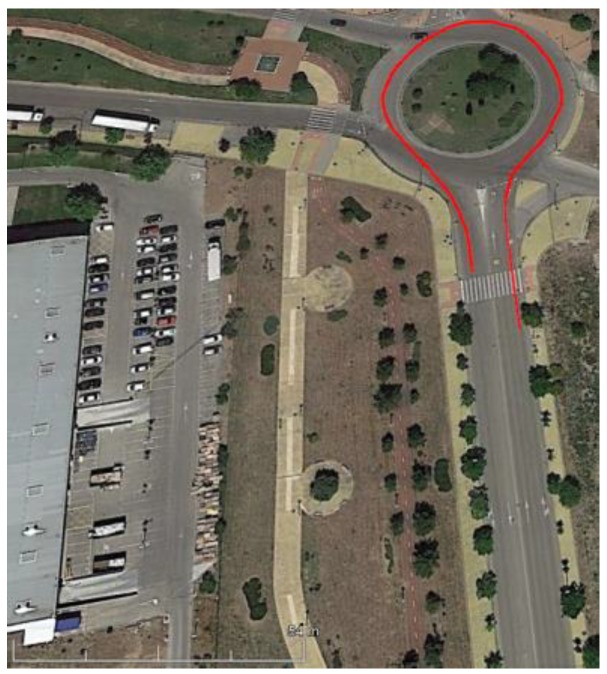
Test 1: Map and vehicle trajectory (Map scale 1:2100 cm).

**Figure 6:**
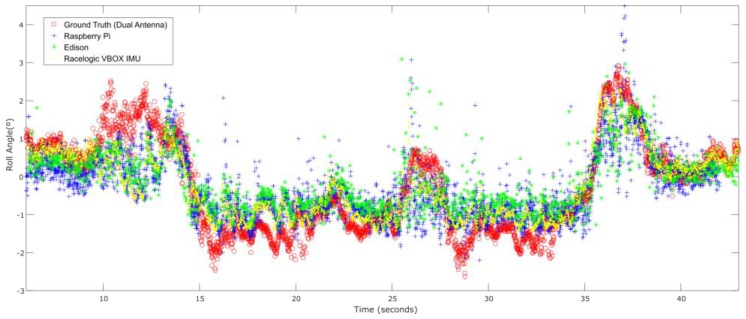
ANN estimated + ground truth roll angle for J-Turn maneuver.

The first test is performed in a roundabout with a radius of 22 m (see Figure 5) at a constant speed (close to 40 Km/h). Figure 6 shows the roll angle estimated by the Raspberry Pi 3 Model B (blue) and the Intel Edison (green). In order to verify estimation accuracy, results were compared with the roll angle measured with the VBOX GPS dual antenna (yellow), which is considered as the ground truth. Estimations are very similar in both devices, and the usage of low-cost devices do not impact ANN estimator performance.

To quantify this impact, the norm, the root mean square (RMS) and maximum errors were calculated. The norm error as a function of time is calculated as follows [7]:

(1) $$E_{t}=\frac{\varepsilon_{t}}{\sigma_{t}}\cdot 100$$

(2) $${\varepsilon_{t}}^{2}=\int_{0}^{T}{\left(\theta_{GT}-\theta_{lc}\right)}^{2}dt\;\sigma_{t}{}^{2}=\int_{0}^{T}{\left(\theta_{GT}-\mu_{GT}\right)}^{2}dt$$

where *θ_GT_* represents the ground truth data, *θ_lc_* represents the low-cost sensor data and *µ_GT_* is the mean value of the ground truth data obtained during the period T.

Table 3 contains the errors measured. To verify the validity of the results, three similar tests for the J-turn maneuver were carried out. To quantify the dispersion of data values, the standard deviation was included for the RMS error (see Table 3). The results show that the error is very similar in both devices and it is higher than the estimated roll angle using VBOX IMU data. The difference between Raspberry Pi 3 Model B and Intel Edison for the norm and RMS error is about 3% and 0.05°, respectively. Concerning maximum errors, the difference is about 0.3°.

An important aspect to consider in this kind of system is the temporal performance and real-time constraints. For the given case, the system needs to be able to process the inputs and apply the ANN estimator in less than 20 ms, corresponding to the sampling rate of 50 Hz forced by the low-cost sensors.

**Figure 7:**
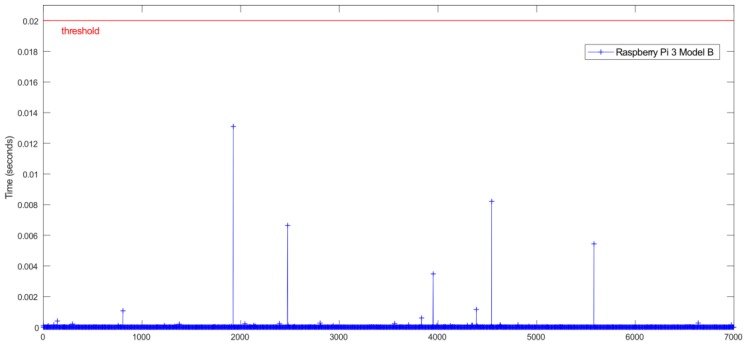
Test 1. Processing time of each iteration for Raspberry Pi 3 Model B.

**Figure 8:**
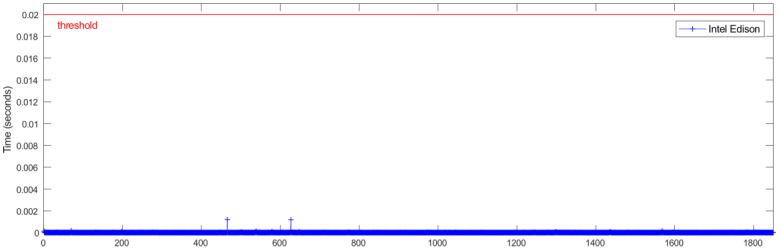
Test 1. Processing time of each iteration for Intel Edison.

Figure 7 and Figure 8 show the relationship between the sensors’ measured data processing time (normalization + ANN estimation + denormalization) for both Intel Edison and Raspberry Pi 3 Model B, respectively, and the established threshold corresponding to the sampling rate (50 Hz).

In Table 4, a comparison of time performance between Intel Edison and Raspberry Pi 3 Model B is presented. To quantify the performance of the devices, the mean and maximum processing times have been calculated. The mean deviation to assess the stability of the devices was also calculated. Results show that both devices estimate roll angle four orders of magnitude lower than the required sample rate threshold of 20 ms. Results show that the processing times for Raspberry Pi 3 Model B are higher than the Intel Edison ones. Concerning the mean and maximum times, the differences are about 0.5 × 10^−3^ ms and 12 ms, respectively. Regarding Mean Deviation, the difference is about 0.008 ms; thus, it is possible to conclude that the results are homogeneous as far as performance and response times are concerned.

### 3.2. Test 2. Double Lane Change

**Figure 9:**
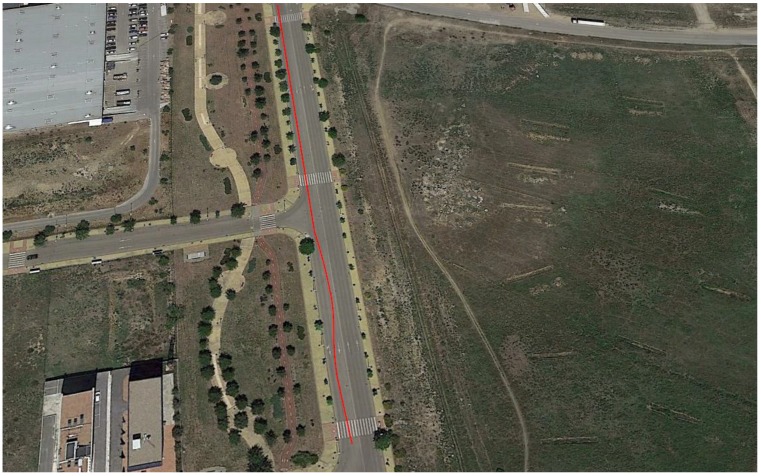
Test 2: Map and vehicle trajectory (Map scale 1:2100 cm).

**Figure 10:**
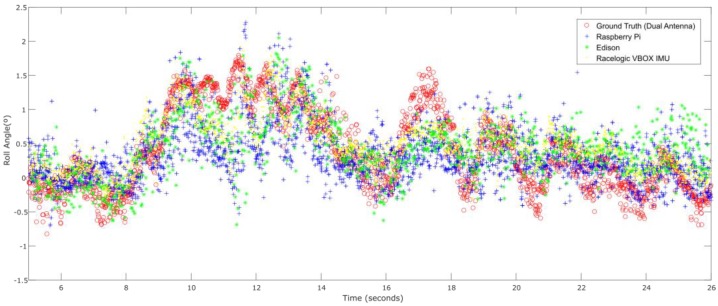
ANN estimated + ground truth roll angle for double lane change.

The second test is carried out in a straight line when the vehicle does a slalom at constant speed (See Figure 9). Figure 10 shows the roll angle estimated by the Raspberry Pi 3 Model B (blue), the Intel Edison (green) and the data provided by the Racelogic IMU (yellow). In order to verify the accuracy of the estimation, they have been compared with the roll angle measured with the GPS dual antenna of VBOX, which is considered as the ground truth. It can be seen that the estimation is very similar in both cases and using low-cost devices do not impact ANN performance.

To quantify this impact, the norm, RMS and maximum errors were calculated (see Table 5). To verify the validity of the results, three similar tests for the Lane Change maneuver were carried out. To quantify the dispersion of data values, the standard deviation were included for RMS error. The results show that the errors are very similar in both devices and they are higher than the estimated roll angle using VBOX IMU data. The difference between Raspberry Pi 3 Model B and Intel Edison for the norm and RMS errors are about 0.6% and 0.03°, respectively. Concerning maximum errors, the difference is about 0.2°.

**Figure 11:**
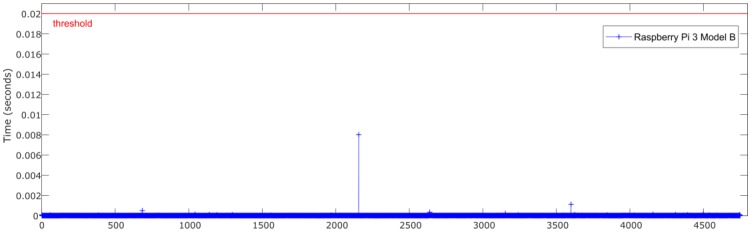
Test 2. Processing time of each iteration for Raspberry Pi 3 Model B.

**Figure 12:**
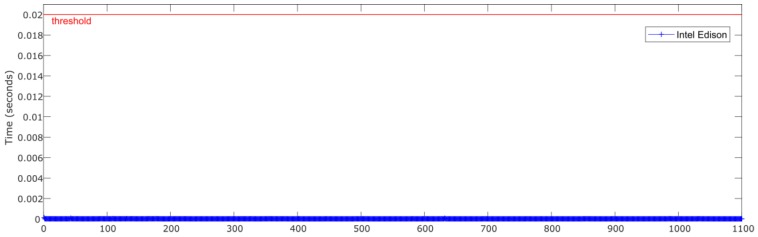
Test 2. Processing time of each iteration for Intel Edison.

Figure 11 and Figure 12 show the relationship between the sensors’ measured data processing time (normalization + ANN estimation + denormalization) for both Intel Edison and Raspberry Pi 3 Model B, respectively, and the established threshold corresponding to the sampling rate (50 Hz).

In Table 6, a time performance comparison between Intel Edison and Raspberry Pi 3 Model B is presented. To quantify this performance for both devices, the mean and maximum processing times were calculated. The mean deviation was also calculated in order to assess the stability of the devices. Results show that both devices estimate roll angle four orders of magnitude lower than the required sample rate threshold of 20 ms. Results show that the processing times for Raspberry Pi 3 Model B are higher than the Intel Edison ones. With regard to mean and maximum times, the difference is about 0.7 × 10^−3^ ms and 7.9 ms. Regarding mean deviation, the difference is about 3.8 × 10^−3^ ms; thus, it is possible to conclude that the results are homogeneous as far as performance and response times are concerned.

### 3.3. Test 3. General Circulation

This last test was carried out in the circuit shown in Figure 4. In this test, not only were J-Turn and Lane Change maneuvers performed, but also the course of a real circuit under usual circulation conditions was covered. The vehicle was driven with the most appropriate speed for the road and the situation, doing severe maneuvers at low and medium speed circulation (between 20 and 60 Km/h), and smooth movements.

**Figure 13:**
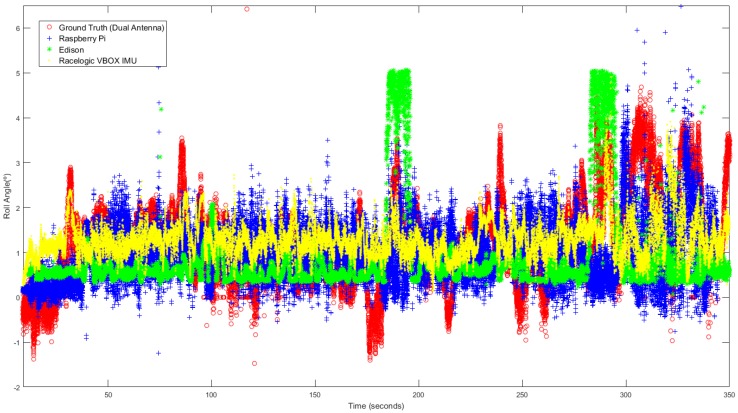
ANN estimated + ground truth roll angle for general circulation.

Figure 13 shows the roll angle estimated by the Raspberry Pi 3 Model B (blue), Intel Edison (green) and the data provided by the Racelogic IMU (yellow), considered as ground truth. In this test, the calculated error is higher than the other two tests. This kind of tests is prone to suffer noise, and as is indicated in [23], the low-cost sensors used are very sensitive to noise. Table 7 shows that the error is higher in Intel Edison than in Raspberry Pi 3 Model B; in this case, Intel Edison presents some atypical data. The difference of the norm and RMS error is about 96% and 0.9°, respectively. Concerning maximum errors, the difference is about 3°.

**Figure 14:**
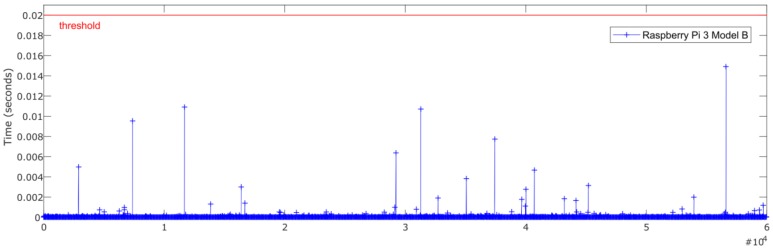
Test 3. Processing time of each iteration for Raspberry Pi 3 Model B.

**Figure 15:**
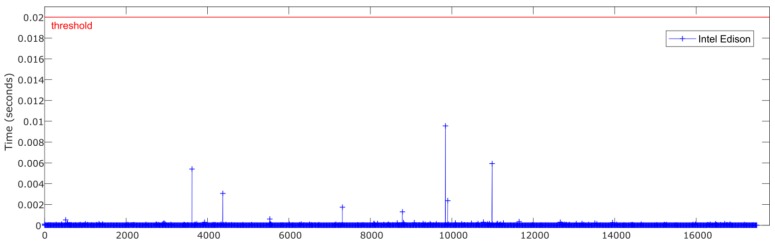
Test 3. Processing time of each iteration for Intel Edison.

Figure 14 and Figure 15 show the relationship between the sensors measured data processing time (normalization + ANN estimation + denormalization) for both Intel Edison and Raspberry Pi 3 Model B, respectively, and the established threshold corresponding to the sampling rate (50 Hz).

In Table 8, a comparison of time performance between Intel Edison and Raspberry Pi 3 Model B is presented. To quantify the performance of the devices, the mean and maximum processing time was calculated. The mean deviation was also calculated in order to assess the stability of the devices. As in previous tests, it was observed that both devices estimate the roll angle four orders of magnitude lower than the required sample rate. Results show that the processing times for Raspberry Pi 3 Model B were higher than the Intel Edison ones. With regard to the mean and maximum times, the difference was about 0.7 × 10^−3^ ms and 5.1 ms, respectively. Regarding Mean Deviation, the difference was about 0.1 × 10^−6^ s; thus, it is possible to conclude that the results are homogeneous as far as performance and response times is concerned.

## 4. Discussion

The following discussion is focused on the precision and performance of the low-cost devices.

### 4.1. Precision

Results show the estimation of the roll angle obtained from low-cost devices is like the measurements directly obtained from Racelogic VBOX. Figure 13 shows that there exists noise in the estimated values for low speed and smooth movements. One reason is that the low-cost sensors are more prone to noise, as is indicated in [23], which negatively affects the ANNs results. This influence of noise explains the high error in the normal circulation test in comparison with the other two kinds of tests. As future work, it is planned to integrate filters via software components to solve the noise-related issue.

Despite the noise influence, the average RMS error in Intel Edison and in Raspberry Pi 3 Model B is 0.8°.

### 4.2. Processing Capability

The temporal performance and real-time constraints are main aspects to consider in order to integrate estimators and controllers in embedded low-cost devices. The results show that the processing time to get the data, execute its normalization, perform the roll angle estimation via ANN and the denormalization of the outcome, is four orders of magnitude lower than the required sample rate threshold of 20 ms. The average mean processing time is 14.5 × 10^−3^ ms for Raspberry Pi 3 Model B and 13 × 10^−3^ ms for Intel Edison. This difference allows to integrate filters in order to reduce the noise in data collected from the sensors as is indicated in 4.1 and to develop and embed more complex estimators and controllers.

To minimize the processing time and allow an objective performance comparison among the low-cost kits, some optimizations were considered before deploying and executing the software components. The most relevant optimizations include:

- Development of same C++ source code for Intel Edison and Raspberry Pi 3 Model B. Usage of same compiler (gcc version 6.3.0) in both platforms, and the same linker and compiler flags, considering the maximum optimization level for speed (-O3 [51]). These additional optimizations perform, among others, predictive commoning optimization, this is, reusing computations (especially memory loads and stores) performed in previous iterations of loops, with beneficial results considering the processor caches in both Intel Edison and Raspberry Pi 3 Model B.
- Usage of light and optimized Fast Artificial Neural Network Library (FANN [47]), version 2, compiled directly in the platforms using *cmake*, and the last source code revision from GitHub [52], that presents among its multiple benefits cache optimization for extra speed.
- Multiple revisions of source code to keep it clean and simple. Algorithmic optimizations to keep a low-profile memory usage, and increased performance (i.e., avoiding copies of objects, like the ANN instance, by passing it by reference).

## 5. Conclusions

In accordance with the results obtained, it can be concluded that low-cost experimental kits including embedded ANN estimators provide roll angle estimations very close to actual values. Even more, Intel Edison and Raspberry Pi 3 Model B have enough computing capabilities to successfully run roll angle estimation based on ANNs to determine rollover risk situations fulfilling real-time operation restrictions stated for this problem.

The results can be used to design, implement, and test an efficient, versatile and scalable low-cost hardware/software architecture able to be integrated in commercial vehicles.

Even more, the performance levels achieved indicate the possibility to embed, in the low-cost experimental kits, more complex estimators using a sensor fusion approach to obtain roll angle estimations closer to the actual values based on Kalman filters, combining neural networks and Kalman filters and considering deep learning techniques, including other parameters obtained from other sources (i.e., road characteristics). In this line, more complex estimators for other variables (such as side slip, etc.) could be integrated in low-cost experimental kits to improve vehicle stability control in real time.

## Figures and Tables

**Table 1:** Technical specifications of hardware elements included in the study.

	VBOX Kit	Raspberry Pi 3 Model B Kit	Intel Edison Kit
RAM	2 GB ^1^	1 GB	1 GB
CPU	Intel Core 2 Duo T8100 2.10 GHz ^1^	4x ARM Cortex-A53, 1.2 GHz	4x Intel Atom Tangier x86 dual core processor +Intel Quark core
Power consumption	Max. 5.5 Watts ^2^	5 V@<1.5 W–6 W	3.3 V@<1 W
Dimensions	170 × 121 × 41 mm^3^	85.60 × 56.5 mm^2^	35.5 × 25 mm^2^
Angular rate range	±150°/s	From ±125°/s to ±2000°/s	From ±245°/s to ±2000°/s
Acceleration range	±1.7 g	From ±2 g to ±16 g	From ±2 g to ±16 g
Angular rate resolution	0.01°/s	16 bits (From 0.003°/s for ±125°/s to 0.06°/s for ±2000°/s)	16 bits (From 0.007°/s for ±245°/s to 0.06°/s for ±2000°/s)
Acceleration resolution	0.01 g	14 bits (From 0.0002 g for ±2 g to 0.002 g for ±16 g)	14 bits (From 0.0002 g for ±2 g to 0.002 g for ±16 g)
Price	>16,000 €	63.2 €	55.5 €

^1^ This information corresponds to the laptop required to control the experiments and register log information in the ground truth kit; ^2^ Only VBOX logger and IMU.

**Table 2:** Experiments proposed.

Id	Description	Times	Purpose	Variables to Observe
1	Vehicle takes a roundabout with a radius of around 20 m at a constant speed near 40 km/h.	3	(1) Estimator Accuracy (2) Estimator Performance	a_ym_, a_x_, $\dot{\psi }$, $\dot{\varphi }$, *φ*_a_, *φ*_e_
2	Vehicle performs a lane change a constant speed near 40 km/h.	3	(1) Estimator Accuracy (2) Estimator Performance	a_ym_, a_x_, $\dot{\psi }$, $\dot{\varphi }$, *φ*_a_, *φ*_e_
3	Vehicle simulates a normal circulation behavior, between 20 and 50 km/h. Several curves were taken, and the vehicle was at the most appropriate speed for the road and conditions	1	(1) Estimator Accuracy (2) Estimator Performance	a_ym_, a_x_, $\dot{\psi }$, $\dot{\varphi }$, *φ*_a_, *φ*_e_

**Table 3:** Test 1. Errors of estimated roll angle on Raspberry Pi 3 Model B and Intel Edison compared with the measured roll from VBOX (ground truth).

	Roll Angle		
	Norm Error (%)	RMS Error (°)	Maximum Error (°)
Raspberry Pi 3 Model B	62.09	0.7405 ± 0.0823	3.54
Intel Edison	65.74	0.7965 ± 0.0743	3.84
Racelogic VBOX IMU	52.22	0.5792 ± 0.0322	2.74

**Table 4:** Test 1. Processing time on Raspberry Pi 3 Model B and Intel Edison.

	Processing Time		
	Maximum (ms)	Mean (ms)	Mean Deviation (ms)
Raspberry Pi 3 Model B	13.09	18.06 × 10^−3^	13.1 × 10^−3^
Intel Edison	1.19	13.87 × 10^−3^	5.1 × 10^−3^

**Table 5:** Test 2. Errors of estimated roll angle on Raspberry Pi 3 Model B and Intel Edison compared with the measured roll from VBOX (ground truth).

	Roll Angle		
	Norm Error (%)	RMS Error (°)	Maximum Error (°)
Raspberry Pi 3 Model B	85.37	0.5302 ± 0.0681	2.54
Intel Edison	85.98	0.5075 ± 0.0432	2.36
Racelogic VBOX IMU	72.84	0.4521 ± 0.0215	1.95

**Table 6:** Test 2. Processing time on Raspberry Pi 3 Model B and Intel Edison.

	Processing Time		
	Maximum (ms)	Mean (ms)	Mean Deviation (ms)
Raspberry Pi 3 Model B	8.02	12.32 × 10^−3^	6.1 × 10^−3^
Intel Edison	0.13	11.59 × 10^−3^	2.3 × 10^−3^

**Table 7:** Test 3. Errors of estimated roll angle on Raspberry Pi 3 Model B and Intel Edison compared with the measured roll from VBOX (ground truth).

	Roll Angle		
	Norm Error (%)	RMS Error (°)	Maximum Error (°)
Raspberry Pi 3 Model B	107.91	1.0321	5.92
Intel Edison	135.87	1.3297	4.41
Racelogic VBOX IMU	92.09	0.9431	5.29

**Table 8:** Test 3. Processing time on Raspberry Pi 3 Model B and Intel Edison.

	Processing Time		
	Maximum (ms)	Mean (ms)	Mean Deviation (ms)
Raspberry Pi 3 Model B	14.88	14.58 × 10^−3^	5.8 × 10^−6^
Intel Edison	9.54	15.09 × 10^−3^	5.9 × 10^−6^

## Abbreviations

The following abbreviations are used in this manuscript:

ANN	Artificial Neural Network
FANN	Fast Artificial Neural Network (software library)
GPS	Global Positioning System
IMU	Inertial Measurement Unit
IoT	Internet Of Things
RMS	Root Mean Square
RSC	Roll Stability Control (system)
